# Supplementary material for: Detailed molecular and epigenetic characterization of the pig IPEC-J2 and chicken SL-29 cell lines
Source: iScience. 2023 Feb 20;26(3):106252. doi: 10.1016/j.isci.2023.106252 (PMC10018572; doi:10.1016/j.isci.2023.106252)
Supplement: Data S2. Complete homer output for identified motifs in Chicken SL-29, related to Tables 5 and 6 — Homer motif analysis results for histone modifications H3K4me1, H3K4me3, H3K27ac, enhancers, and ATAC-seq of chicken SL-29 cell line. Parameters for possible false positives is as mentioned earlier for S5. [file mmc3.zip › Data_S2/S6/Chicken_SL_29/peak_files_CTCF/homerResults.html]

peak\_files\_CTCF/ - Homer de novo Motif Results


# Homer *de novo* Motif Results (peak\_files\_CTCF/)

Known Motif Enrichment Results  
Gene Ontology Enrichment Results  
If Homer is having trouble matching a motif to a known motif, try copy/pasting the matrix file into
STAMP  
More information on motif finding results: HOMER
| Description of Results
| Tips
  
Total target sequences = 29483  
Total background sequences = 29106  
\* - possible false positive  

|  |  |  |  |  |  |  |  |  |
| --- | --- | --- | --- | --- | --- | --- | --- | --- |
| Rank | Motif | P-value | log P-pvalue | % of Targets | % of Background | STD(Bg STD) | Best Match/Details | Motif File |
| 1 | T C A G G A C T C A T G G C T A A T C G G C A T G T A C G T C A A G C T G A T C | 1e-1238 | -2.852e+03 | 18.96% | 6.06% | 80.9bp (87.8bp) | Fra1(bZIP)/BT549-Fra1-ChIP-Seq(GSE46166)/Homer(0.992) More Information \| Similar Motifs Found | motif file (matrix) |
| 2 | A T G C A G T C T C G A A G T C C G A T A C G T A T G C A T G C A C T G T A C G | 1e-356 | -8.207e+02 | 30.60% | 20.60% | 81.2bp (93.7bp) | Fli1(ETS)/CD8-FLI-ChIP-Seq(GSE20898)/Homer(0.964) More Information \| Similar Motifs Found | motif file (matrix) |
| 3 | A G T C A C T G A G T C A G T C G T A C A G T C A G T C A C G T T A C G A T C G A G C T A C T G A C T G | 1e-259 | -5.972e+02 | 4.39% | 1.43% | 73.0bp (95.3bp) | BORIS(Zf)/K562-CTCFL-ChIP-Seq(GSE32465)/Homer(0.945) More Information \| Similar Motifs Found | motif file (matrix) |
| 4 | A T G C A T G C A C T G G T A C G T A C T G C A A C G T A T G C C A G T A G C T | 1e-184 | -4.254e+02 | 6.07% | 2.85% | 82.0bp (98.4bp) | YY2/MA0748.2/Jaspar(0.918) More Information \| Similar Motifs Found | motif file (matrix) |
| 5 | C G T A T C G A T G A C G T A C C T G A A C G T T G C A A G C T A C T G A C G T | 1e-179 | -4.136e+02 | 25.87% | 19.06% | 83.3bp (86.8bp) | TCF4(bHLH)/SHSY5Y-TCF4-ChIP-Seq(GSE96915)/Homer(0.957) More Information \| Similar Motifs Found | motif file (matrix) |
| 6 | A G C T T C G A G A C T A C T G C T G A A G T C C T A G G A C T T G A C C G T A | 1e-140 | -3.243e+02 | 8.59% | 5.06% | 79.0bp (92.5bp) | Atf1(bZIP)/K562-ATF1-ChIP-Seq(GSE31477)/Homer(0.976) More Information \| Similar Motifs Found | motif file (matrix) |
| 7 | A T G C C T A G T A G C C G T A C G A T A C G T G A T C A G T C | 1e-109 | -2.521e+02 | 9.56% | 6.20% | 83.0bp (91.0bp) | TEAD1(TEAD)/HepG2-TEAD1-ChIP-Seq(Encode)/Homer(0.946) More Information \| Similar Motifs Found | motif file (matrix) |
| 8 | C T A G C A T G T A C G T C G A T G C A C G T A A G C T A C G T T G A C G T A C | 1e-98 | -2.273e+02 | 5.54% | 3.16% | 84.0bp (88.4bp) | NFkB-p65-Rel(RHD)/ThioMac-LPS-Expression(GSE23622)/Homer(0.901) More Information \| Similar Motifs Found | motif file (matrix) |
| 9 | A G C T G C A T G C T A C T A G T G A C A C T G A T G C A T C G | 1e-70 | -1.624e+02 | 26.10% | 21.72% | 84.5bp (99.2bp) | E2F4(E2F)/K562-E2F4-ChIP-Seq(GSE31477)/Homer(0.703) More Information \| Similar Motifs Found | motif file (matrix) |
| 10 | A G T C A G T C A G C T G T C A C T G A T C A G T A C G T A C G T A G C A T C G | 1e-69 | -1.590e+02 | 15.59% | 12.12% | 84.7bp (92.8bp) | AP-2alpha(AP2)/Hela-AP2alpha-ChIP-Seq(GSE31477)/Homer(0.735) More Information \| Similar Motifs Found | motif file (matrix) |
| 11 | T A G C A T C G A T C G A T G C A T C G A C G T A C G T A G C T A G C T A G C T | 1e-68 | -1.578e+02 | 44.74% | 39.71% | 84.8bp (95.8bp) | PB0131.1\_Gmeb1\_2/Jaspar(0.582) More Information \| Similar Motifs Found | motif file (matrix) |
| 12 | A G T C T C A G T C A G A T G C A G T C T G C A C T G A A C G T T G A C T C A G | 1e-63 | -1.464e+02 | 4.62% | 2.84% | 81.9bp (92.2bp) | NFY(CCAAT)/Promoter/Homer(0.905) More Information \| Similar Motifs Found | motif file (matrix) |
| 13 | A T C G A G T C A C T G A C T G A G T C A G T C A C G T C T A G | 1e-55 | -1.278e+02 | 5.86% | 3.95% | 85.0bp (100.2bp) | Zfx/MA0146.2/Jaspar(0.791) More Information \| Similar Motifs Found | motif file (matrix) |
| 14 | A T G C C T G A A G T C A C T G C G A T A C T G C T G A A G T C | 1e-53 | -1.235e+02 | 5.94% | 4.05% | 83.9bp (90.6bp) | TFEC/MA0871.2/Jaspar(0.981) More Information \| Similar Motifs Found | motif file (matrix) |
| 15 | G A C T T A C G A G T C C T A G A G T C T C G A C G A T A C T G A G T C A C T G A G C T T C G A A T G C | 1e-44 | -1.016e+02 | 1.42% | 0.66% | 82.3bp (106.3bp) | NRF(NRF)/Promoter/Homer(0.847) More Information \| Similar Motifs Found | motif file (matrix) |
| 16 | C G T A C A G T G A T C G C A T C A T G A T C G G A C T A C G T A G C T A G C T T G C A G T C A C A G T | 1e-41 | -9.645e+01 | 1.16% | 0.50% | 83.3bp (89.5bp) | Pitx1:Ebox(Homeobox,bHLH)/Hindlimb-Pitx1-ChIP-Seq(GSE41591)/Homer(0.756) More Information \| Similar Motifs Found | motif file (matrix) |
| 17 | A G T C G A C T A C G T G C T A G T C A C T G A T C A G T A C G A T C G T A C G T A G C G T A C T C A G | 1e-41 | -9.484e+01 | 0.61% | 0.18% | 85.4bp (93.1bp) | TATA-Box(TBP)/Promoter/Homer(0.606) More Information \| Similar Motifs Found | motif file (matrix) |
| 18 | A C T G T G A C C A T G C T A G G T C A A G T C A C G T C G T A A G T C T C G A C T A G A G T C A C G T | 1e-33 | -7.812e+01 | 0.13% | 0.01% | 73.1bp (0.0bp) | THAP11/MA1573.1/Jaspar(0.615) More Information \| Similar Motifs Found | motif file (matrix) |
| 19 | C G T A C G T A A G T C A G T C A C G T C G T A A G T C A T G C A T G C A T C G | 1e-30 | -6.940e+01 | 0.12% | 0.01% | 77.8bp (37.3bp) | PB0161.1\_Rxra\_2/Jaspar(0.719) More Information \| Similar Motifs Found | motif file (matrix) |
| 20 | T A C G T A G C A T G C A C G T T G A C A G C T A T G C T A C G A T G C A T C G C T G A A T C G T C G A | 1e-29 | -6.880e+01 | 0.46% | 0.14% | 71.6bp (103.5bp) | GFX(?)/Promoter/Homer(0.927) More Information \| Similar Motifs Found | motif file (matrix) |
| 21 | G A C T C G A T C A T G A T C G G T C A C G A T C G A T C A T G A G T C G C A T C A G T G C A T T C G A | 1e-28 | -6.655e+01 | 0.12% | 0.01% | 78.3bp (76.1bp) | DUX4(Homeobox)/Myoblasts-DUX4.V5-ChIP-Seq(GSE75791)/Homer(0.658) More Information \| Similar Motifs Found | motif file (matrix) |
| 22 | A C G T G T A C A C T G C G T A C G T A A G T C A G T C A G T C C T A G A C T G | 1e-27 | -6.329e+01 | 0.16% | 0.02% | 82.6bp (47.9bp) | PB0034.1\_Irf4\_1/Jaspar(0.630) More Information \| Similar Motifs Found | motif file (matrix) |
| 23 | A C T G G A C T G A C T T A C G A G T C G A T C T G C A A G C T C T A G C T A G T A G C C T G A T C G A | 1e-24 | -5.665e+01 | 0.67% | 0.29% | 80.3bp (91.3bp) | Rfx1(HTH)/NPC-H3K4me1-ChIP-Seq(GSE16256)/Homer(0.978) More Information \| Similar Motifs Found | motif file (matrix) |
| 24 | G T C A A C T G A T G C A C T G A T G C G T A C A G T C G T C A A G C T C G T A A C T G A G T C A C T G | 1e-14 | -3.424e+01 | 0.06% | 0.00% | 84.1bp (69.7bp) | PB0127.1\_Gata6\_2/Jaspar(0.559) More Information \| Similar Motifs Found | motif file (matrix) |
| 25 \* | C A G T G C A T C G T A A T C G C A T G A T C G G C A T C G A T C T G A C T A G C T A G C A T G C G A T | 1e-10 | -2.482e+01 | 0.13% | 0.04% | 83.2bp (47.9bp) | PSE(SNAPc)/K562-mStart-Seq/Homer(0.694) More Information \| Similar Motifs Found | motif file (matrix) |
